# Toll-Like Receptor 4 Mediates Methamphetamine-Induced Neuroinflammation through Caspase-11 Signaling Pathway in Astrocytes

**DOI:** 10.3389/fnmol.2017.00409

**Published:** 2017-12-12

**Authors:** Si-Hao Du, Dong-Fang Qiao, Chuan-Xiang Chen, Si Chen, Chao Liu, Zhoumeng Lin, Huijun Wang, Wei-Bing Xie

**Affiliations:** ^1^School of Forensic Medicine, Southern Medical University, Guangzhou, China; ^2^Guangzhou Forensic Science Institute, Guangzhou, China; ^3^Department of Anatomy and Physiology, Institute of Computational Comparative Medicine (ICCM), College of Veterinary Medicine, Kansas State University, Manhattan, KS, United States

**Keywords:** methamphetamine, Caspase-11, Toll-like receptor 4 (TLR4), neuroinflammation, inflammasome, astrocyte

## Abstract

Methamphetamine (METH) is an amphetamine-typed stimulant drug that is increasingly being abused worldwide. Previous studies have shown that METH toxicity is systemic, especially targeting dopaminergic neurons in the central nervous system (CNS). However, the role of neuroinflammation in METH neurotoxicity remains unclear. We hypothesized that Toll-like receptor 4 (TLR4) and Caspase-11 are involved in METH-induced astrocyte-related neuroinflammation. We tested our hypothesis by examining the changes of TLR4 and Caspase-11 protein expression in primary cultured C57BL/6 mouse astrocytes and in the midbrain and striatum of mice exposed to METH with western blot and double immunofluorescence labeling. We also determined the effects of blocking Caspase-11 expression with wedelolactone (a specific inhibitor of Caspase-11) or siRNA on METH-induced neuroinflammation in astrocytes. Furthermore, we determined the effects of blocking TLR4 expression with TAK-242 (a specific inhibitor of TLR4) or siRNA on METH-induced neuroinflammation in astrocytes. METH exposure increased Caspase-11 and TLR4 expression both *in vitro* and *in vivo*, with the effects *in vitro* being dose-dependent. Inhibition of Caspase-11 expression with either wedelolactone or siRNAs reduced the expression of inflammasome NLRP3 and pro-inflammatory cytokines. In addition, blocking TLR4 expression inhibited METH-induced activation of NF-κB and Caspase-11 *in vitro* and *in vivo*, suggesting that TLR4-Caspase-11 pathway is involved in METH-induced neuroinflammation. These results indicate that Caspase-11 and TLR4 play an important role in METH-induced neuroinflammation and may be potential gene targets for therapeutics in METH-caused neurotoxicity.

## Background

Methamphetamine (METH) is one of the most widely abused drugs and the most commonly synthesized illegal drug worldwide (Kiyatkin and Sharma, 2009; Cai et al., 2016; Li et al., 2017). A major target of METH is the central nervous system (CNS), especially the central dopaminergic circuitries (Carmena et al., 2015; Huang et al., 2015; Chen et al., 2016; Mendieta et al., 2016). Previous studies have demonstrated that short-term exposure to METH can cause widespread brain damage and long-term exposure to METH can lead to CNS neurodegeneration (Wu et al., 2014; Andres et al., 2015). Accumulating evidence suggests that METH exposure can alter the functions of glial cells and activate astrocytes (Granado et al., 2011; Loftis and Janowsky, 2014; Fernandes et al., 2016; Bortell et al., 2017). Activated astrocytes play important roles in METH-induced neurotoxicity (Hebert and O’Callaghan, 2000; Abdul Muneer et al., 2011; Carmena et al., 2015). However, the underlying mechanisms of how astrocytes mediate METH-induced neurotoxicity remain to be elucidated.

Astrocytes represent the largest population of glial cells in the CNS and have a variety of functions, including maintenance of brain homeostasis, storage of energy substrates, and as a major component of the blood-brain barrier and the synapses (Liu et al., 2017; Singh and Abraham, 2017). Neuroinflammation is a process mediated by microglia, astrocytes, neurons, T cells, neutrophils, mast cells and inflammatory mediators released from these cells (Shabab et al., 2017). Astrocytes can mediate neuroinflammation by secreting specific signaling molecules, such as pro-inflammatory cytokines and anti-inflammatory cytokines, including interleukin (IL), interferon (IFN), chemokines and tumor necrosis factor (Whitney et al., 2009; Granado et al., 2011). Activation of microglia and astrocytes is initially a normal compensatory neuroinflammatory response to brain injury, but excessive neuroinflammation can lead to further brain damage (Whitney et al., 2009; Kempuraj et al., 2016; Pal et al., 2016).

Toll-like receptors (TLRs) are a class of immunological pattern recognition receptors (Krawczyk-Michalak et al., 2008; Shirjang et al., 2017). In mammals, 13 TLRs have been identified that can recognize different endogenous ligands or exogenous pathogens from protozoa, bacteria, fungi or viruses. Toll-like receptor 4 (TLR4) is one of the most widely studied receptors in the TLR family because it is the only one that can active both of the following two pathways: Myd88-dependent and non Myd88-dependent pathways (Billod et al., 2016). In the Myd88-dependent pathway, Myd88 induces the downstream tumor necrosis factor receptor-associated factor 6, interleukin-1 receptor-associated kinase (IRAK) activation and then induces NF-κB activation, thereby mediating inflammatory and pro-inflammatory cytokine production (Shen et al., 2016). In the non Myd88-dependent pathway, TLR4 triggers the activation of the TRIF-related adaptor molecule (TRAM), followed by the activation of interferon regulatory factor 3, which induces the production of IFN-γ. In the later inflammatory response process, TRAM can also activate NF-κB, inducing inflammatory factor expression and activation (Brempelis et al., 2017; Yan et al., 2017).

Caspase-11 is a member of the caspase family, which is a group of structurally related cysteine proteases (Roberts and Yilmaz, 2015). Caspase-11 plays an important role in apoptosis, inflammation and cell migration (Coutermarsh-Ott et al., 2016; Zanoni et al., 2016). In our previous study, we found that neuronal apoptosis in METH-treated rats was associated with Caspase-11 activation (Huang et al., 2015). Other studies have shown that Caspase-11 can be induced by lipopolysaccharides (LPS) secreted by gram-negative bacteria, leading to inflammation, and this reaction is closely related to the activation of Caspase-1 (Kayagaki et al., 2015). Caspase-1 and inflammasome adaptor protein apoptosis-associated speck-like protein containing CARD (ASC) together participate in the assembly of inflammasomes, and mediate the expression of downstream inflammatory factors. Previous studies have shown that the activation of inflammasome nucleotide-binding oligomerization domain-like receptor family pyrin domain containing 3 (NLRP3) is mediated by the activation of Caspase-1, which promotes the pro-inflammatory cytokine secretion (Meng et al., 2014; Zhu et al., 2016). Therefore, specific inhibition of inflammasome NLRP3 activation pathway maybe is a potential strategy to the therapeutic treatment of related diseases. Inflammatory cytokines and inflammation play an important role in many diseases. Our previous work and other studies have confirmed that METH induces dopamine neuronal damage through apoptosis, autophagy, oxidative stress and other mechanisms (Krasnova and Cadet, 2009; Abdul Muneer et al., 2011; Qiao et al., 2014; Wu et al., 2014). However, the role of glial cells and its possible molecular mechanisms in METH neurotoxicity are still unclear, which is important and warrants further study.

The objective of this study was to investigate the role of TLR4 and Caspase-11 in METH-induced neuroinflammation. To this end, we determined changes of TLR4 and Caspase-11 expression and the levels of inflammatory factors in primary cultured astrocytes, and the corpus striatum and midbrain of mice exposed to METH. We found that METH exposure increased Caspase-11 and TLR4 expression; inhibition of Caspase-11 or TLR4 reduced Caspase-1 and ASC activation and pro-inflammatory cytokine production *in vitro* and *in vivo*. Our results indicate that both TLR4 and Caspase-11 play a crucial role in METH-induced neuroinflammation and these proteins may be potential therapeutic targets for neuronal injury caused by METH.

## Materials and Methods

### Materials

Cell culture reagents, including Dulbecco’s modified Eagle’s medium/F12 (DMEM/F12) medium, fetal bovine serum (FBS) and trypsin were purchased from Gibco (Carlsbad, CA, USA). METH (>99% purity) was obtained from the National Institutes for the Control of Pharmaceutical and Biological Products (Beijing, China). Anti-Myd88, anti-Caspase-1 and anti-rabbit and mouse IgG (H + L), F(ab’)2 fragment (Alexa Fluor 555 conjugate) were purchased from the Cell Signaling Technology (Boston, MA, USA). Anti-GFAP was purchased from Arigo Biolaboratories. Anti-NF-κB, anti-TLR4, anti-IL-1β, anti-TIRAP, anti-TRIF and anti-IL-18 were purchased from ABclonal Inc. (College Park, MD, USA). Anti-β-actin and goat anti-mouse and rabbit IgG (H + L)-HRP were purchased from Beijing Ray Antibody Biotech (Beijing, China). Anti-NLRP3, anti-Caspase-11 and anti-ASC were purchased from Bioss (Beijing, China). Fluorescein (FITC)-conjugated goat anti-mouse and rabbit IgG were purchased from DingGuo (Beijing, China). siRNAs for TLR4, Myd88, TRIF, TIRAP, Caspase-11 and NF-κB were purchased from the Shanghai GenePharma Company Limited (Shanghai, China). TAK-242 (C_15_H_17_ClFNO_4_S; a specific inhibitor of TLR4) was purchased from Sigma-Aldrich (St.Louis, MO, USA). Wedelolactone (Wed; a specific inhibitor of Caspase-11) was purchased from Aladdin (Shanghai, China). Super ECL Assay was purchased from KeyGEN Biotech (Nanjing, China). Other chemicals or reagents, unless specifically mentioned below, were purchased from Sigma-Aldrich (St. Louis, MO, USA).

### Animal Protocol

Healthy adult male C57BL/6 mice (18–22 g, 6–8 weeks old) were purchased from Laboratory Animal Center of Southern Medical University (Guangzhou, China) and were singly housed in tub cages in a temperature-controlled (approximately 22°C) room with a 12 h light/dark cycle. Animal care and experimental procedures were approved by the Institutional Animal Care and Use Committee at the Southern Medical University and followed the latest NIH Guidelines for the Care and Use of Laboratory Animals (NIH in 2011). The animals were habituated to the animal facility for 1 week before use. The mice were divided randomly into four groups (*n* = 3/group): saline control group, METH subacute exposure group, TAK-242 exposure group and METH + TAK-242 exposure group. METH was dissolved in saline. The mice in the subacute exposure group received eight intraperitoneal (i.p.) injections of METH (15 mg/kg/injection) at 12 h intervals. This exposure paradigm was chosen based on our and other previous studies to mimic human METH abuse (Cadet et al., 2003; Krasnova and Cadet, 2009; Qiao et al., 2014; Xu et al., 2017). This exposure paradigm is relevant to human exposure because the measured concentrations of METH in the blood and brain (~0.1–1.1 μg/ml in blood and ~0.4–1.8 μg/g in brain; Table 1) of mice at 2 h after the last injection were in the range of reported blood concentrations in METH abusers (0.6–5 μg/ml [4–30 μM]; Winek et al., 2001; Huang et al., 2015). The saline control group (vehicle group) mice received a similar volume of physiological 0.9% saline via i.p. injections according to the same schedule as the subacute exposure group. TAK-242 was initially dissolved in DMSO, and then further diluted in saline. In the TAK-242 exposure group, TAK-242 was administered i.p. once per day for 5 days (3 mg/kg/injection; Fang et al., 2014). In the METH + TAK-242 exposure group, TAK-242 was given i.p. daily for 5 days, and beginning from the second day METH was given i.p. for eight injections at 12 h intervals. All animals survived throughout the study period. Mice were euthanized (CO_2_; followed by decapitation) at 2 h after the last injection. Brain samples were rapidly removed, and the midbrain and striatum were dissected on an ice-cold glass plate, rapidly frozen and stored at −80°C until analysis.

**Table 1:** Methamphetamine (METH) concentrations in the blood and brain of vehicle-treated and METH-exposed mice at 2 h after the last injection.

Control			METH-treated		
No	Blood (ng/ml)	Brain (ng/g)	No	Blood (ng/ml)	Brain (ng/g)
1	<0	<0	1	110	397.04
2	<0	<0	2	660	528.24
3	<0	<0	3	1050	1797.82

### METH Concentrations in Mice Brain and Blood

Blood and brain samples of vehicle-treated and METH-treated mice were collected at 2 h after the last injection. Brains were homogenated in PBS. Then, we measured METH concentrations using a previously reported LC-MS/MS (AB4000Q, USA) protocol and the concentrations were calculated based on a standard curve (Huang et al., 2015).

### Cell Culture

Primary astrocytes were cultured in DMEM/F12 medium supplemented with 10% FBS, 50 units/ml penicillin G, and 50 mg/ml streptomycin sulfate at 37°C in a humidified atmosphere of 5% CO_2_. The cells were passaged every 6 days. Isolation and identification of mouse primary astrocytes were performed as previously described (Zhang et al., 2017a,b).

### METH and Inhibitor Treatment

Once cells reached about 80% in 6-well plates, medium was changed to non-serum medium and cells were exposed to 0, 0.5, 1.0, 1.5, 2.0 or 2.5 mM METH in primary astrocytes for 24 h. This concentration range was selected based on the results of LC25 (data not shown), and this concentration is similar to the concentrations used in other studies (Huang et al., 2009; Cisneros and Ghorpade, 2014; Zhang et al., 2015; Cao et al., 2016). According to the western blot results, at the 2.0 mM METH treatment, the expressions of IL-1β and IL-18 were the highest. In the experiments with inhibitors, the cells were pre-cultured for 3 h with 100 nM TAK-242 or 30 μM wedelolactone and then incubated with 2.0 mM METH for 24 h. The concentrations of TAK-242 and wedelolactone were selected based on earlier studies (Ii et al., 2006; Matsunaga et al., 2011; Huang et al., 2015) and the results of LC25 (data not shown), and these concentrations had optimal inhibition effects in our experimental model.

### siRNA and Transfection

Small interfering RNA (siRNA) was synthesized by GenePharma (Shanghai, China). The sequences of siRNA are shown in Table 2. Primary astrocytes were seeded onto a 6-well plate (4 × 10^5^ cells/well). When cells reached 80% confluence, 5 μl Lipofectamine 3000 (Invitrogen, Carlsbad, CA, USA) reagent and 20 μmol siRNA or siNC were added in opti-MEM medium (Gibco BRL, Paisley, UK). The mixed solution was incubated at room temperature for 20 min, and then siRNA mixture was added gently and slowly in each well, and then 1 ml complete medium was added in each well. After 6 h incubation, all supernatant was discarded and then 2 ml complete medium was added in each well.

**Table 2:** The sequences of small interfering RNAs (siRNAs) used in the present study.

Gene	Number	The sequence of siRNA (5′–3′)
*TLR4*	1	GCAUAGAGGUAGUUCCUAA TT
*Myd88*	1	CCUUUACAGGUGGCCA GAGUGGAAA
*Myd88*	2	GGUCCAUUGCCAGCGAGCUAAUUGA
*NF-κB*	1	GAAGAUUCAUCUGGGUGAAGAUUUA
*TRIF*	1	CCACGUCCUACACGGAAG AUGAUUU
*TRIF*	2	UCUAUCGCAUGAGACAUCAUUACAA
*Caspase-11*	1	GGAACAGCUGGGCAAAGAATT
*Caspase-11*	2	CCACCAUGGUGAAGCUAAUTT
*ASC*	1	GCUACUAUCUGGAGUCGUATT
*ASC*	2	CCCUUGCACAGCCUAUCUUTT
*NC*	1	UUCU CCGAACGUGUCACGUTT

### Western Blot Analysis

Primary cultured astrocytes and brain samples from mice exposed to vehicle or METH were lysed in ice-cold RIPA buffer with protease inhibitors. Protein concentrations were determined with the BCA-100 Protein Quantitative Analysis kit (Biocolors, Shanghai, China). Protein samples were separated by 10–15% sodium dodecyl sulfate polyacrylamide gel (SDS-PAGE) and transferred onto 0.22 μm polyvinylidenedifluoride (PVDF) membranes (Millipore, Billerica, MA, USA). The membranes were incubated at room temperature for 2 h in 5% nonfat milk blocking buffer. After blocking, membranes were incubated with primary antibodies overnight at 4°C (1:500–1000). After the membranes were washed three times with TBST, they were incubated with an anti-rabbit or mouse IgG horseradish peroxidase (1:10,000) for 1 h at room temperature. The membranes were developed with Super ECL Western blotting detection reagents. The signal of band intensities was quantitated by Gel-Pro analyzer (Media Cybernetics, Inc., Rockville, MD, USA). Expression of the housekeeping gene β-actin was used as a reference control.

### Double Immunofluorescence Labeling

To determine TLR4 and NF-κB expression levels in primary cultured astrocytes and mouse midbrain samples, we performed double immunofluorescence labeling on cells and frozen sections of adult mouse midbrains. For immunolabeling, all incubation solutions were prepared using PBS supplemented with 10% normal goat serum and 0.05% Triton X-100. These antibodies were used together with DAPI nuclear labeling. The frozen tissue sections were incubated with blocking buffer (10% BSA in PBS) for 30 min at room temperature, with the primary antibody (anti-GFAP dilution of 1:500, anti-TLR4 dilution of 1:100, or anti-NF-κB dilution of 1:100) overnight at 4°C, and then with the secondary antibody for 1 h at room temperature (FITC conjugated anti-mouse or rabbit IgG dilution of 1:50, Alexa Fluor 555 conjugated anti-mouse or rabbit IgG dilution of 1:200). Microphotographs were taken using fluorescence microscopy (A1+/A1R+; Nikon). All digital images were processed using the same settings to improve the contrast.

### Immunohistochemistry

Brain tissue samples were fixed in 4% formalin, embedded in paraffin and sectioned at 3 μm thickness. Brain sections were treated with xylene to remove the paraffin and then were rehydrated. Prior to staining heat-induced antigen retrieval was performed by placing the slides into 0.01 M citrate buffer solution (pH6.0), and subjected to microwave heating three times for 5 min. Then the sections were incubated with 3% H_2_O_2_ for 10 min at room temperature and washed three times with PBS, followed by incubation with serum for 30 min. Samples were incubated with the indicated primary antibodies (e.g., anti-GFAP dilution of 1:500) overnight at 4°C. After washing with PBS, the slices were incubated with secondary antibodies for 0.5 h at 37°C. After staining with 3,3′-diaminobenzidine (DAB), the sections were observed under optical microscope.

### Statistical Analysis

Data given in the text are expressed as mean ± standard deviation (SD) of at least three independent replicates. Data were analyzed with Student’s *t*-test, one-way analysis of variance (ANOVA), 2 × 2 factorial ANOVA or two-way ANOVA (as appropriate) followed by LSD *post hoc* analyses using SPSS 20.0 software (IBM Corporation, Armonk, NY, USA). The value of *P* < 0.05 was considered statistically significant.

## Results

### Methamphetamine Increases the Expression of Proinflammatory Cytokines IL-1β and IL-18 in Astrocytes

**Figure 1:**
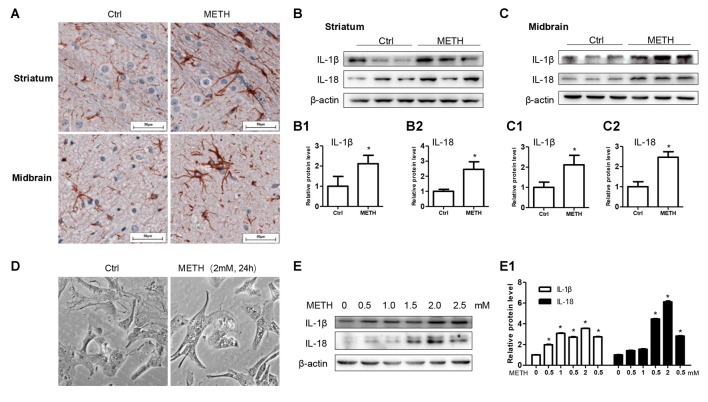
Astrocytes are activated after Methamphetamine (METH) exposure *in vitro* and *in vivo*. Male C57BL/6 mice were divided randomly into control and experiment groups (*n* = 5). Animals were injected intraperitoneally with saline or METH (15 mg/kg/injection, 8 injections, at 12 h intervals). **(A)** Immunohistochemical staining of GFAP in the striatum and midbrain. Scale bar, 50 μm. Western blot **(B,C)** and quantitative analyses **(B1–C2)** were performed to determine IL-1β and IL-18 protein expression in the striatum and midbrain. Primary cultured astrocytes were exposed to 0.5, 1.0, 1.5, 2.0 and 2.5 mM METH for 24 h. **(D)** Morphological changes were observed. Western blot **(E)** and quantitative analyses **(E1)** were performed to determine IL-1β and IL-18 protein expression. β-actin was used as a loading control. Fold induction relative to vehicle-treated group is shown. *p < 0.05 vs. vehicle-treated group. Data were analyzed with Student’s *t*-test or one-way analyses of variance (ANOVA) followed by LSD *post hoc* analysis. Data are expressed as mean ± standard deviation (SD).

To determine whether astrocytes are activated after METH exposure, a mouse model treated with METH (8 injections, 15 mg/kg/injection, at 12 h intervals) was used. Immunohistochemistry staining results showed the size of astrocyte cell body and the number of bulges were increased in the striatum and midbrain, suggesting that METH treatment activates astrocytes (Figure 1A). In order to further examine the inflammatory response of the mouse brain after the treatment of METH, we used the inflammatory indicators of IL-1β and IL-18. IL-1β and IL-18 are proinflammatory cytokines that play a major role in the inflammatory response *in vivo*. Western blot results revealed that the expressions of IL-1β (*t*_(4)_ = 3.027, *P* = 0.0389) and IL-18 (*t*_(4)_ = 2.799, *P* = 0.0489) were increased in the corpus striatum (Figures 1B–B2) of METH-exposed mice compared to the control group. The expressions of IL-1β (*t*_(4)_ = 3.603, *P* = 0.0227) and IL-18 (*t*_(4)_ = 6.825, *P* = 0.0024) were also increased in the midbrain (Figures 1C–C2). Furthermore, the morphology of primary cultured astrocytes was significantly changed after METH exposure. Specifically, vacuole-like secretory vesicles were observed in the cell body of METH-treated astrocytes (Figure 1D). IL-1β (*F*_(1,17)_ = 1966, *P* < 0.0001) and IL-18 (*F*_(1,17)_ = 5616, *P* < 0.0001) protein expression was also significantly increased in a dose-dependent manner in primary cultured astrocytes (Figures 1E,E1). Taken together, these data demonstrated that METH exposure activates astrocytes and induces the expression of proinflammatory factors *in vivo* and *in vitro*.

### Caspase-11 Mediates IL-1β and IL-18 Expression in METH-Exposed Astrocytes

**Figure 2:**
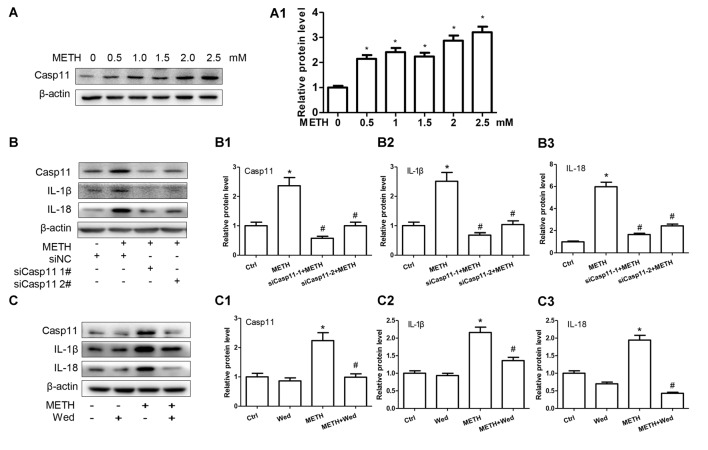
Caspase-11 mediates IL-1β and IL-18 expression in METH-exposed astrocytes. **(A,A1)** Primary cultured astrocytes were exposed to 0.5, 1.0, 1.5, 2.0 and 2.5 mM METH for 24 h. **(B–B3)** Primary cultured astrocytes were transfected with siRNAs targeting Caspase-11 or control siRNA for 24 h followed by METH (2 mM) treatment for 24 h. **(C–C3)** Primary cultured astrocytes were exposed to Wed (30 μM) for 2 h prior to METH (2 mM) treatment as indicated. Western blot **(A–C)** and quantitative analyses were performed to determine Caspase-11, IL-1β and IL-18 protein expression. All the experiments were repeated three times. Data are expressed as mean ± SD. *p < 0.05 vs. non-METH-treated group. ^#^*p* < 0.05 vs. the scrambled + METH treated group. Data in **(A)** were analyzed with one-way ANOVA followed by LSD *post hoc* analyses; data in **(B)** were analyzed with two-way ANOVA followed by LSD *post hoc* analyses; data in **(C)** was analyzed with 2 × 2 factorial ANOVA followed by LSD *post hoc* analyses.

To assess the role of Caspase-11 in METH-caused neuroinflammation in astrocytes, primary cultured astrocytes were exposed to METH for 24 h with or without siCaspase-11. Western blot results showed that the expression level of Caspase-11 was increased by METH in a dose-dependent manner (Figure 2A). Caspase-11 protein level was 2.8-fold higher in the 2.0 mM METH-treated primary cultured astrocytes than in the control (group effect: *F*_(1,17)_ = 20.62, *P* < 0.0001; METH 2.0 mM: *t*_(3)_ = 7.916, *P* < 0.001; Figure 2A1). This increase was normalized after co-treatment with either one of the siCaspase-11 s (*F*_(1,11)_ = 63.638, *P* < 0.001; mean difference of siRNA#1 = 1.7850, *P* < 0.001; mean difference of siRNA#2 = 1.3632, *P* < 0.001; Figures 2B,B1). These results suggest that both of the two siRNAs can effectively knockdown Caspase-11 expression.

Next, we evaluated whether silencing of Caspase-11 can reduce the expression of METH-induced proinflammatory cytokines in astrocytes. Western blot results showed that IL-1β (*F*_(1,11)_ = 63.068, *P* < 0.001; mean difference of siRNA#1 = 1.8308, *P* < 0.001; mean difference of siRNA#2 = 1.4752, *P* < 0.001; Figure 2B2) and IL-18 (*F*_(1,11)_ = 90.282, *P* < 0.001; mean difference of siRNA#1 = 4.3131, *P* < 0.001; mean difference of siRNA#2 = 3.5472, *P* < 0.001; Figure 2B3) expression was significantly decreased after Caspase-11 knockdown in METH-treated primary cultured astrocytes. To confirm this result, we also used Wed, a specific inhibitor of Caspase-11, to block Caspase-11 expression and then examined the expression of IL-1β and IL-18. The expression of Caspase-11 was significantly decreased (*F*_(1,11)_ = 33.394, *P* < 0.001). Consistent with the above-described results, Wed inhibited the expression of IL-1β (*F*_(1,11)_ = 13.470, *P* < 0.001) and IL-18 (*F*_(1,11)_ = 56.288, *P* < 0.001) induced by METH (Figures 2C–C3). These results suggest that METH exposure induces Caspase-11 protein expression and its activation is involved in METH-caused increased expression of IL-1β and IL-18.

### Inflammasome NLRP3 Is Involved in Caspase-11 Mediated Neuroinflammation Signaling Pathways Caused by METH in Astrocytes

**Figure 3:**
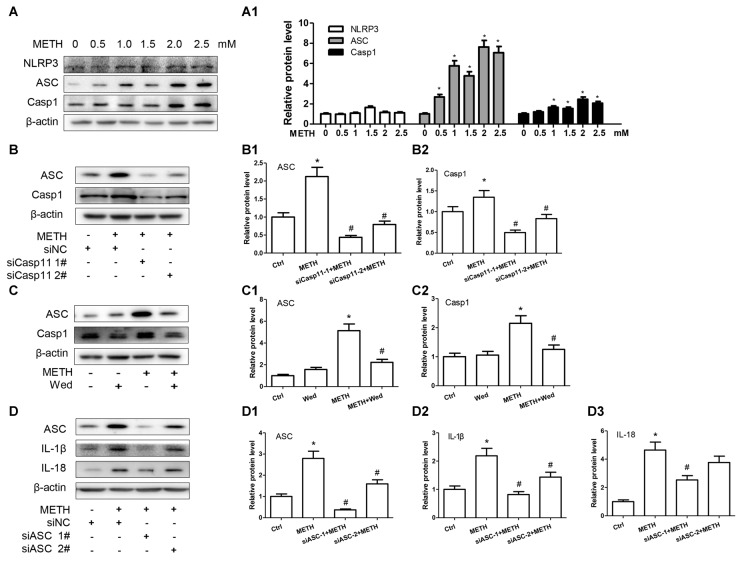
Inflammasomes are involved in Caspase-11-mediated IL-1β and IL-18 protein expression caused by METH in astrocytes. **(A,A1)** Primary cultured astrocytes were exposed to 0.5, 1.0, 1.5, 2.0 and 2.5 mM METH for 24 h. **(B–B2)** Cells were transfected with siRNAs targeting Caspase-11 or control siRNA for 24 h followed by METH (2 mM) treatment for 24 h. **(C–C2)** Cells were exposed to Wed (30 μM) for 2 h prior to METH (2 mM) treatment as indicated. **(D–D3)** Cells were transfected with siRNAs targeting apoptosis-associated speck-like protein containing CARD (ASC) or control siRNA for 24 h followed by METH (2 mM) treatment for 24 h. Western blot **(A–D)** and quantitative analyses were performed to determine Caspase-11, NLRP3, ASC, Caspase-1, IL-1β and IL-18 protein expression. All the experiments were repeated three times. Data are expressed as mean ± SD. *p < 0.05 vs. non-METH-treated group. ^#^*p* < 0.05 vs. the scrambled + METH treated group. Data in **(A)** were analyzed with one-way ANOVA followed by LSD *post hoc* analyses; data in **(B,D)** were analyzed with two-way ANOVA followed by LSD *post hoc* analyses; data in **(C)** were analyzed with 2 × 2 factorial ANOVA followed by LSD *post hoc* analyses.

Previous studies demonstrated that Caspase-11 could mediate the expression of IL-1β and IL-18 via NLRP3/ASC/Caspase-1 signal axis (Zanoni et al., 2016). To examine whether the NLRP3/ASC/Caspase-1 complex is involved in Caspase-11- mediated METH-induced IL-1β and IL-18 expression, we determined the changes on NLRP3, ASC and Caspase-1 protein levels in METH-treated and untreated primary cultured astrocytes by Western blot analysis. The results showed that the expression level of NLRP3 was not changed significantly (*F*_(1,17)_ = 2.753, *P* = 0.0699), but the expression levels of ASC (*F*_(1,17)_ = 30.61, *P* < 0.0001) and Caspase-1 (*F*_(1,17)_ = 13.05, *P* = 0.002) were increased in a dose-dependent manner (Figures 3A,A1).

To further assess whether elevation of ASC and Caspase-1 is involved in Caspase-11-mediated induction of IL-1β and IL-18 by METH, we used siRNAs or specific inhibitor (Wed) to block Caspase-11 expression and then observed the expression changes on ASC and Caspase-1 before and after Caspase-11 silencing. Western blot results showed decreased expression of Caspase-1 and ASC after Caspase-11 expression knockdown (Caspase-1: *F*_(1,11)_ = 69.870, *P* < 0.001; mean difference of siRNA#1 = 1.6883, *P* < 0.001; mean difference of siRNA#2 = 1.3302, *P* < 0.001; ASC: *F*_(1,11)_ = 27.891, *P* < 0.001; mean difference of siRNA#1 = 0.8521, *P* < 0.001; mean difference of siRNA#2 = 0.5150, *P* < 0.001) or inhibitor (Caspase-1: *F*_(1,11)_ = 72.187, *P* < 0.001; ASC: *F*_(1,11)_ = 22.810, *P* = 0.001) in METH-treated primary cultured astrocytes (Figures 3B–B2,C–C3). In addition, we used siRNAs targeting ASC to silence ASC expression and then examined the effects on METH-caused neuroinflammation in primary cultured astrocytes. Western blot results showed that both of siRNAs could effectively knockdown ASC expression, with siRNA #1 being more effective (*F*_(1,11)_ = 77.319, *P* < 0.001; mean difference of siRNA#1 = 2.4278, *P* < 0.001; mean difference of siRNA#2 = 1.2030, *P* = 0.001). The ASC silence significantly decreased the expression of IL-1β (*F*_(1,11)_ = 36.266, *P* < 0.001; mean difference of siRNA#1 = 1.3684, *P* < 0.001; mean difference of siRNA#2 = 0.7536, *P* = 0.005) and IL-18 (*F*_(1,11)_ = 48.380, *P* < 0.001; mean difference of siRNA#1 = 2.1178, *P* = 0.001; mean difference of siRNA#2 = 0.8849, *P* = 0.151; Figures 3D–D3). Taken together, these results suggest that Caspase-11 mediates METH-induced neuroinflammation through NLRP3/ASC/Caspase-1 signaling pathway in astrocytes.

### TLR4 Is Necessary for METH-Induced IL-1β and IL-18 Expression in Astrocytes

**Figure 4:**
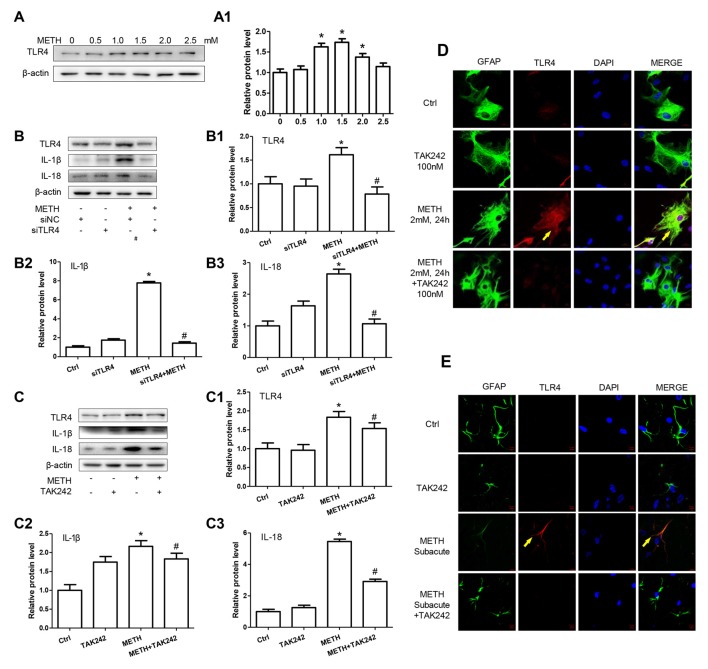
Toll-like receptor 4 (TLR4) is necessary for METH-induced IL-1β and IL-18 expression in astrocytes. **(A,A1)** Primary cultured astrocytes were exposed to 0.5, 1.0, 1.5, 2.0 and 2.5 mM METH for 24 h. **(B–B3)** Cells were transfected with siRNAs targeting TLR4 or control siRNA for 24 h followed by METH (2 mM) treatment for 24 h. **(C–C3,D)** Cells were exposed to TAK-242 (100 nM) for 2 h prior to METH (2 mM) treatment. Western blot **(A–C)** and quantitative analyses were performed to determine TLR4, IL-1β and IL-18 protein expression. Immunolabeling and confocal imaging analysis **(D)** showed elevated TLR4 expression in the cells treated with METH compared with controls. **(E)** Male C57BL/6 mice were divided randomly into control, METH, TAK-242 and METH + TAK-242 groups (*n* = 3/group). TAK-242 was injected intraperitoneally with DMSO and saline (3 mg/kg/injection, five injections, at 24 h intervals). At the 2nd day, animals were injected intraperitoneally with saline or METH (15 mg/kg/injection, eight injections, at 12 h intervals). Midbrain tissues were harvested at 2 h after the last dosing. Immunolabeling and confocal imaging analysis showed elevated TLR4 expression in the midbrain of METH-exposed mice compared with controls. Yellow arrow refers to TLR4. Cell experiments were repeated three times. Data are expressed as mean ± SD. *p < 0.05 vs. non-METH-treated group. ^#^*p* < 0.05 vs. the scrambled + METH treated group. Data in **(A)** were analyzed with one-way ANOVA followed by LSD *post hoc* analyses; data in **(B,C)** were analyzed with 2 × 2 factorial ANOVA followed by LSD *post hoc* analyses.

TLR4, a receptor located in the cell membrane, is the first line of defense of innate immunity system. In the present study, we found that TLR4 protein expression was increased after treatment with METH in primary cultured astrocytes in a dose-dependent manner (*F*_(1,17)_ = 12.41, *P* < 0.001; Figures 4A,A1). After silencing TLR4 with siRNA (*F*_(1,11)_ = 20.278, *P* = 0.002), the METH-caused increased expression of IL-1β (*F*_(1,11)_ = 1678.59, *P* < 0.001) and IL-18 (*F*_(1,11)_ = 162.589, *P* < 0.001) was substantially ameliorated (Figures 4B–B3). To confirm these results, we also used a specific inhibitor of TLR4, bromomethyl acetate (TAK-242), to inhibit the expression of TLR4 and then examined the effect on expression of IL-1β and IL-18 in astrocytes. Western blot (Figures 4C,C1) and immunofluorescence staining (Figure 4D) results showed that TAK-242 pretreatment attenuated METH-induced TLR4 expression in primary cultured astrocytes (*F*_(1,11)_ = 24.026, *P* < 0.001). Similar results were observed in the midbrain of METH-treated C57BL/6 mice with or without TAK-242 co-treatment (Figure 4E). Consistent with the results of silencing TLR4 expression by siRNA, we observed that inhibition of TLR4 expression by TAK-242 also reduced the expression of IL-1β (*F*_(1,11)_ = 38.922, *P* < 0.001) and IL-18 (*F*_(1,11)_ = 260.665, *P* < 0.001) induced by METH in astrocytes (Figures 4C2,C3). These results suggest that METH exposure induces TLR4 protein expression and this activation is involved in the METH-induced expression of IL-1β and IL-18 in astrocytes.

### Caspase-11 Is Involved in TLR4-Mediated IL-1β and IL-18 Expression in METH-Exposed Astrocytes

**Figure 5:**
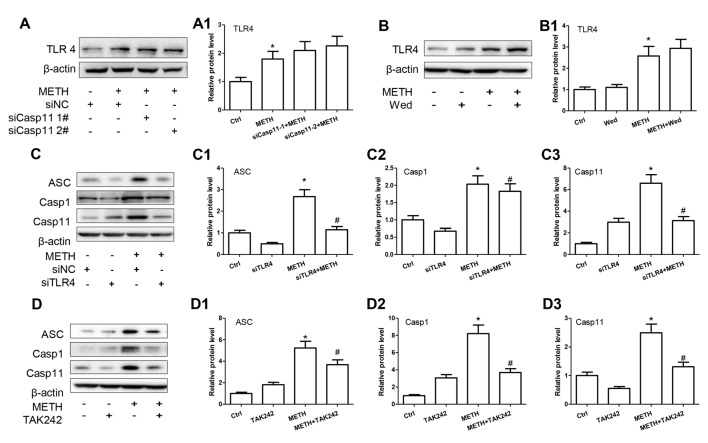
Caspase-11 is involved in TLR4-mediated IL-1β and IL-18 expression in METH-exposed astrocytes. Primary cultured astrocytes transfected with siRNAs targeting Caspase-11 **(A,A1)**, TLR4 **(C–C3)** or control siRNA for 24 h followed by METH (2 mM) treatment for 24 h. Cells were exposed to Wed (30 μM) **(B,B1)** or TAK-242 (100 nM) **(D–D3)** for 2 h prior to METH (2 mM) treatment as indicated. Western blot **(A–D)** and quantitative analyses were performed to determine TLR4, Caspase-11, ASC and Caspase-1 protein expression. All the experiments were repeated three times. Data are expressed as mean ± SD. *p < 0.05 vs. non-METH-treated group. ^#^*p* < 0.05 vs. the scrambled + METH treated group. Data in **(A)** were analyzed with two-way ANOVA followed by LSD *post hoc* analyses; data in **(B–D)** were analyzed with 2 × 2 factorial ANOVA followed by LSD *post hoc* analyses.

According to the above-described results, both Caspase-11 and TLR4 mediate METH-induced expression of IL-1β and IL-18 in astrocytes, but the relation between Caspase-11 and TLR4 is still not clear. To address this, we determined the effect on TLR4 expression after blocking Caspase-11 expression by siRNAs or specific inhibitor targeting Caspase-11. Western blot results showed that blockade of Caspase-11 expression by siRNAs (*F*_(1,11)_ = 12.158, *P* = 0.002; mean difference of siRNA#1 = −0.3007, *P* > 0.05; mean difference of siRNA#2 = −0.4628, *P* > 0.05) or Wed (*F*_(1,11)_ = 2.185, *P* = 0.178) had no effects on TLR4 expression (Figures 5A,A1,B,B1), suggesting that TLR4 is not the downstream target of Caspase-11. Conversely, we found that METH-induced expression of Caspase-11 was significantly attenuated after TLR4 silencing by siRNAs (*F*_(1,11)_ = 97.975, *P* < 0.001) or TAK-242 (*F*_(1,11)_ = 12.284, *P* < 0.001; Figures 5C,C3,D,D3), which indicates that TLR4 can regulate Caspase-11 expression. Notably, we also observed that the expression of ASC and Caspase-1 were decreased significantly after blockade of TLR4 expression by siRNA (ASC: *F*_(1,11)_ = 22.297, *P* = 0.001; Caspase-1: *F*_(1,11)_ = 39.431, *P* < 0.001) or TAK-242 (ASC: *F*_(1,11)_ = 25.985, *P* < 0.001; Caspase-1: *F*_(1,11)_ = 98.882, *P* < 0.001; Figures 5C1,C2,D1,D2). These results indicate that TLR4 mediates METH-induced expression of IL-1β and IL-18 through Caspase-11/ASC/Caspase-1 signal axis in astrocytes.

### TLR4 Induces Translocation of NF-κB into the Nucleus, Leading to an Increase of Caspase-11 Transcription in METH-Exposed Astrocytes

**Figure 6:**
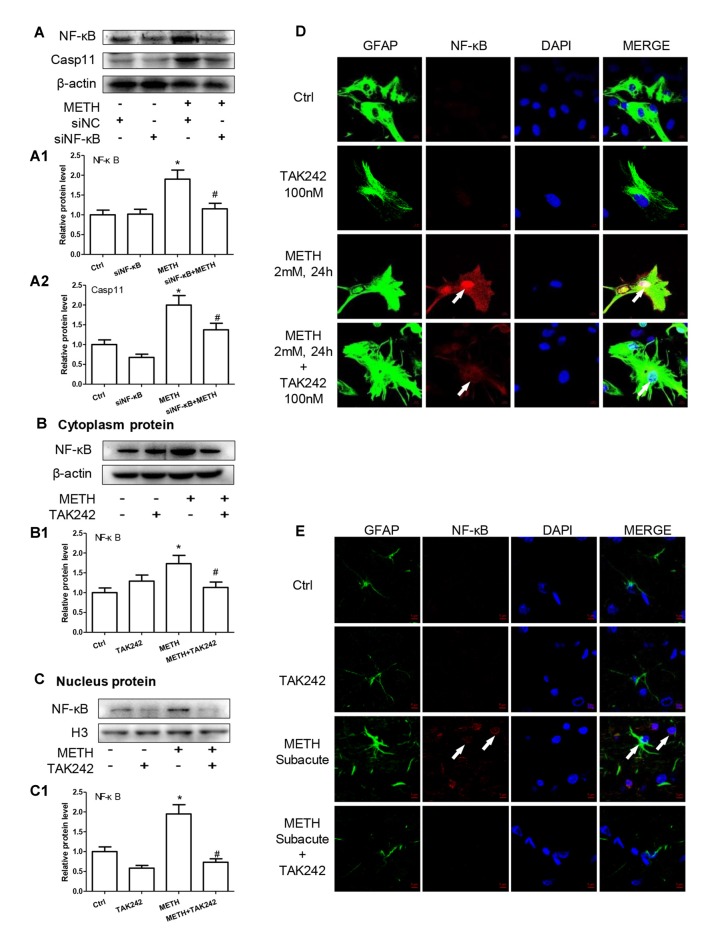
TLR4 induces translocation of NF-κB into the nucleus, leading to an increase of Caspase-11 transcription in METH-exposed astrocytes. **(A–A2)** Primary cultured astrocytes were transfected with siRNAs targeting NF-κB or control siRNA for 24 h followed by METH (2 mM) treatment for 24 h. **(B–D)** Cells were exposed to TAK-242 (100 nM) for 2 h prior to METH (2 mM) treatment as indicated, nucleus proteins **(C,C1)** and cytoplasm **(B,B1)** proteins were extracted separately. Western blot **(A–C)** and quantitative analyses were performed to determine NF-κB, and Caspase-11 protein expression. H3 was used as the loading control of nucleus proteins. Immunolabeling and confocal imaging analysis **(D)** showed elevated NF-κB expression in the METH-treated cells compared with controls. **(E)** The animal exposure paradigms were the same as described in Figure 4. Immunolabeling and confocal imaging analysis showed elevated NF-κB expression in the midbrain of METH-exposed mice compared with controls. White arrow refers to NF-κB. Cell experiments were repeated three times. Data are expressed as mean ± SD. *p < 0.05 vs. non-METH-treated group. ^#^*p* < 0.05 vs. the scrambled + METH treated group. Data were analyzed with 2 × 2 factorial ANOVA followed by LSD *post hoc* analyses.

We have demonstrated that TLR4 mediates METH-induced expression of IL-1β and IL-18 via Caspase-11-mediated Caspase-1-dependent pathway. The next question is to clarify how TLR4 regulates the expression of Caspase-11. Previous studies have shown that NF-κB, a well-known downstream target of TLR4, can bind to promoter of Caspase-11 and activate Caspase-11 expression (Dolunay et al., 2017). We hypothesized that TLR4 regulates the expression of Caspase-11 through NF-κB signaling axis. To test this hypothesis, we used siRNA targeting to NF-κB to silence the expression of NF-κB and then determined the effect on Caspase-11 expression in astrocytes after METH exposure. The results showed that siRNA can effectively knockdown METH-induced NF-κB expression (*F*_(1,11)_ = 17.625, *P* = 0.003), and METH-induced expression of Caspase-11 was significantly decreased after silencing NF-κB (*F*_(1,11)_ = 36.680, *P* < 0.001; Figures 6A–A2). This result suggests that NF-κB regulates METH-caused increased expression of Caspase-11. To further clarify the mechanisms of how TLR4 regulates the expression of Caspase-11 via NF-κB, we observed the location changes of NF-κB after METH exposure with or without TAK-242 pre-treatment. We extracted nucleus and cytoplasm proteins separately from METH-treated astrocytes with or without TAK-242 pre-treatment, and then measured NF-κB protein level in the nucleus and cytoplasm. Western blot results showed that METH exposure increased the expression of NF-κB both in the cytoplasm (*F*_(1,11)_ = 23.819, *P* < 0.001) and nucleus (*F*_(1,11)_ = 23.606, *P* < 0.001); and this effect was significantly mitigated by co-treatment with TAK-242 (Figures 6B,B1,C,C1). To confirm the results, we also performed the immunofluorescence staining. Results showed that METH exposure increased the expression of NF-κB, particularly in the nucleus, and this effect was significantly mitigated by pre-treatment with the TAK-242 (Figure 6D). Similar results were observed in the midbrain of METH-treated C57BL/6 mice with or without TAK-242 pre-treatment (Figure 6E). Taken together, these results suggest that TLR4 regulates the expression of Caspase-11 via NF-κB.

### TLR4 Mediates METH-Induced IL-1β and IL-18 Expression through Both Myd88-Dependent and Myd88-Independent Signaling Pathways

**Figure 7:**
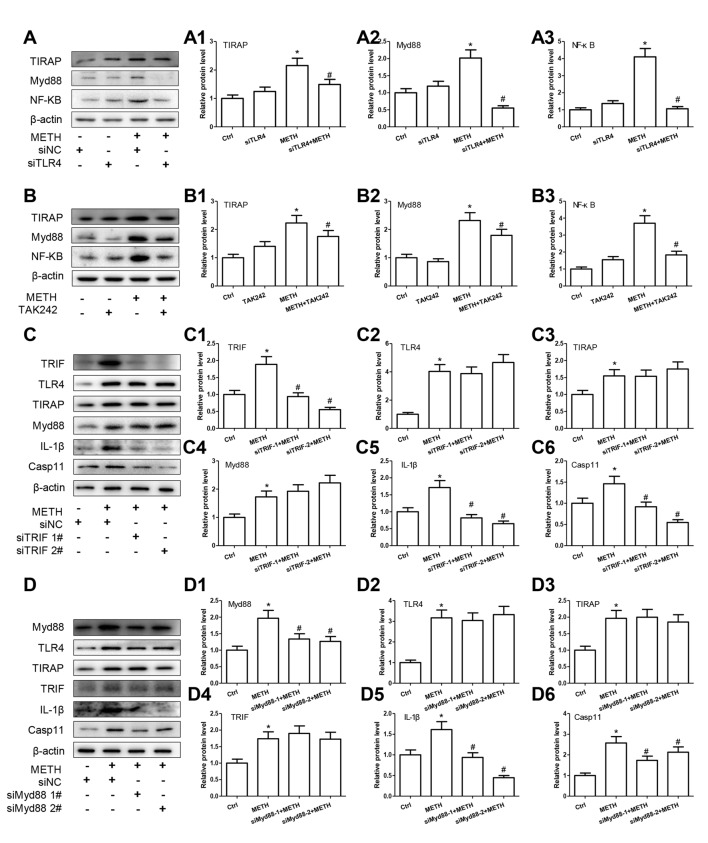
TLR4 mediates METH-induced IL-1β and IL-18 expression through both Myd88-dependent and Myd88-independent signaling pathways. Primary cultured astrocytes transfected with siRNAs targeting TLR4 **(A–A3)**, TRIF **(C–C6)**, Myd88 **(D–D6)** or control siRNA for 24 h followed by METH (2 mM) treatment for 24 h. Cells were exposed to TAK-242 (100 nM) **(B–B3)** for 2 h prior to METH (2 mM) treatment as indicated. Western blot **(A–D)** and quantitative analyses were performed to determine TLR4, TRIF, TIRAP, Myd88, NF-κB, Caspase-11 and/or IL-1β protein expression. All the experiments were repeated three times. Data are expressed as mean ± SD. *p < 0.05 vs. non-METH-treated group. ^#^*p* < 0.05 vs. the scrambled + METH treated group. Data in **(A,B)** were analyzed with 2 × 2 factorial ANOVA followed by LSD *post hoc* analyses; data in **(C,D)** were analyzed with two-way ANOVA followed by LSD *post hoc* analyses.

TLR4 regulates the NF-κB expression through two-independent signal axes: one is TRIF signaling axis and the other is TIRAP/Myd88/IRAK4 signal axis (Van Acker et al., 2014; Planès et al., 2016). To explore whether TLR4 regulates NF-κB expression through TIRAP/Myd88/IRAK4 axis and/or TRIF axis in METH-exposed astrocytes, we used siRNA and TAK-242 to silence TLR4 expression and then examined the protein levels of TIRAP, Myd88 and NF-κB. Western blot results showed that TRIF, Myd88 and NF-κB protein levels were significantly increased in METH-treated astrocytes compared with control cells and then significantly decreased after inhibiting TLR4 expression by siRNA (TIRAP: *F*_(1,11)_ = 18.088, *P* = 0.003; Myd88: *F*_(1,11)_ = 83.986, *P* < 0.001; NF-κB: *F*_(1,11)_ = 116.232, *P* < 0.001) or TAK-242 (TIRAP: *F*_(1,11)_ = 14.603, *P* < 0.001; Myd88: *F*_(1,11)_ = 37.914, *P* < 0.001; NF-κB: *F*_(1,11)_ = 59.806, *P* < 0.001; Figures 7A–A3,B–B3), suggesting that both TRIF axis and TIRAP/Myd88/IRAK4 axis were activated in METH-treated astrocytes.

To assess whether activated TRIF axis and TIRAP/Myd88/IRAK4 axis are involved in TLR4-mediated METH-induced expression of IL-1β and IL-18, we designed siRNAs targeting TRIF and Myd88, respectively, to inhibit their expression and block the two pathways. For TRIF axis, Western blot analysis showed that both of two siRNAs can effectively knockdown METH-induced TRIF expression (*F*_(1,11)_ = 46.137, *P* < 0.001; mean difference of siRNA#1 = 0.9489, *P* < 0.001; mean difference of siRNA#2 = 1.3343, *P* < 0.001), and as we expected, silencing of TRIF expression had no effect on the expression of TLR4 (*F*_(1,11)_ = 41.028, *P* < 0.001; mean difference of siRNA#1 = 0.1537, *P* > 0.05; mean difference of siRNA#2 = −0.6282, *P* = 0.716), TIRAP (*F*_(1,11)_ = 9.750, *P* = 0.005; mean difference of siRNA#1 = 0.0093, *P* > 0.05; mean difference of siRNA#2 = −0.2144, *P* > 0.05), and Myd88 (*F*_(1,11)_ = 17.865, *P* = 0.001; mean difference of siRNA#1 = −0.1990, *P* > 0.05; mean difference of siRNA#2 = −0.4939, *P* = 0.131), but significantly decreased the expression of Caspase-11 (*F*_(1,11)_ = 27.421, *P* < 0.001; mean difference of siRNA#1 = 0.5428, *P* = 0.001; mean difference of siRNA#2 = 0.9130, P *=* < 0.001) and IL-1β (*F*_(1,11)_ = 36.249, *P* < 0.001; mean difference of siRNA#1 = 0.8943, *P* < 0.001; mean difference of siRNA#2 = 1.064, *P* < 0.001; Figures 7C–C6). These results indicate that TRIF can regulate the expression of IL-1β through Caspase-11.

For TIRAP/Myd88/IRAK4 axis, the two Myd88 siRNAs can effectively knockdown METH-induced Myd88 expression (*F*_(1,11)_ = 16.972, *P* = 0.001; mean difference of siRNA#1 = 0.6301, *P* = 0.012; mean difference of siRNA#2 = 0.7037, *P* = 0.006), as shown in Figures 7D–D6. Furthermore, we found that silencing Myd88 expression inhibited METH-induced expression of Caspase-11 (*F*_(1,11)_ = 24.584, *P* < 0.001; mean difference of siRNA#1 = 0.8441, *P* = 0.002; mean difference of siRNA#2 = 0.4460, *P* = 0.048) and IL-1β (*F*_(1,11)_ = 46.602, *P* < 0.001; mean difference of siRNA#1 = 0.6738, *P* = 0.001; mean difference of siRNA#2 = 1.1653, *P* < 0.001), while had no effect on the expression of TLR4 (*F*_(1,11)_ = 31.889, *P* < 0.001; mean difference of siRNA#1 = 0.1242, *P* > 0.05; mean difference of siRNA#2 = −0.1581, *P* > 0.05), TRIAP (*F*_(1,11)_ = 15.216, *P* = 0.001; mean difference of siRNA#1 = −0.032, *P* > 0.05; mean difference of siRNA#2 = 0.1140, *P* > 0.05) and TRIF (*F*_(1,11)_ = 14.190, *P* = 0.001; mean difference of siRNA#1 = 0.9489, *P* < 0.001; mean difference of siRNA#2 = 1.3343, *P* < 0.001). These results indicate that the Myd88-dependent downstream signaling pathway of TLR4 might also be the pathway through which Caspase-11 mediates the expression of inflammatory factors. These results suggest that both TRIF axis and TIRAP/Myd88/IRAK4 axis can regulate Caspase-11 expression and lead to increased expression of IL-1β after METH exposure.

### Silencing of TLR4 Expression Reduces METH-Induced IL-1β and IL-18 Expression *in Vivo*

**Figure 8:**
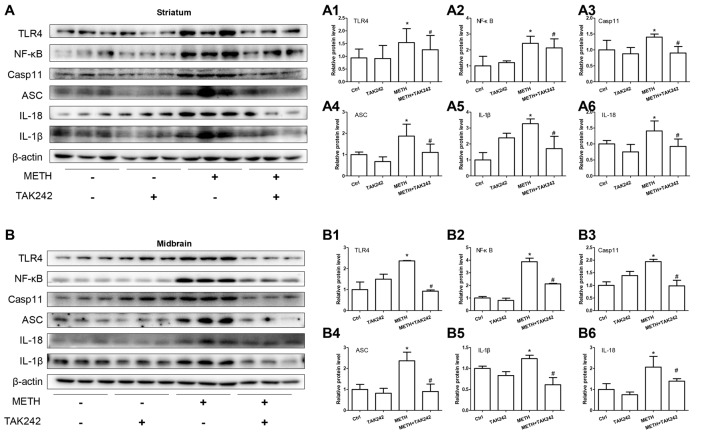
Silencing of TLR4 expression reduces METH-induced IL-1β and IL-18 expression *in vivo*. Mice were exposed to saline vehicle, METH, TAK-242, or METH + TAK-242 as described in the methods section and in Figure 4 legend. Striatum and midbrain tissues were harvested at 24 h after the last dosing. Western blot **(A,B)** and quantitative analyses **(A1–A6,B1–B6)** were performed to determine TLR4, NF-κB, Caspase-11, ASC, IL-18 and IL-1β protein expression. Data are expressed as mean ± SD. *p < 0.05 vs. non-METH-treated group. ^#^*p* < 0.05 vs. METH-treated group. Data were analyzed with 2 × 2 factorial ANOVA followed by LSD *post hoc* analyses.

To confirm the role of TLR4 in METH-induced IL-1β and IL-18 expression *in vivo*, TAK-242, a specific inhibitor of TLR4, was injected to the mice to inhibit TLR4 expression in the brain. After TAK-242 pre-treatment, mice were treated with saline or METH (*n* = 3/group). Western blot analysis showed METH exposure significantly induced TLR4 protein expression; and this effect was substantially attenuated by pre-treatment with TAK-242 in the striatum (*F*_(1,11)_ = 8.407, *P* = 0.02) and midbrain (*F*_(1,11)_ = 80.867, *P* < 0.001; Figures 8A,A1,B,B1). Previously, we demonstrated that IL-1β and IL-18 were regulated by TLR4 *in vitro* (Figures 4B,C). Next, we explored whether silencing TLR4 can decrease METH-induced neuroinflammation in the mouse striatum and midbrain. We measured IL-1β and IL-18 protein expression in each treatment group. We found that METH-induced expression of IL-1β (striatum: *F*_(1,11)_ = 6.752, *P* = 0.032; midbrain: *F*_(1,11)_ = 13.907, *P* = 0.006) and IL-18 (striatum: *F*_(1,11)_ = 23.073, *P* = 0.001; midbrain: *F*_(1,11)_ = 6.8389, *P* = 0.031) both were decreased after pre-treatment with TAK-242 (Figures 8A5,A6,B5,B6). These results were consistent with those *in vitro*. To further affirm the relationship of Caspase-11 and TLR4, NF-κB and ASC protein expression was measured with Western blot. Decreased expression after inhibiting TLR4 expression with TAK-242 was detected for NF-κB (striatum: *F*_(1,11)_ = 7.736, *P* = 0.024; midbrain: *F*_(1,11)_ = 47.831, *P* < 0.001), Caspase-11 (striatum: *F*_(1,11)_ = 6.361, *P* = 0.036; midbrain: *F*_(1,11)_ = 97.043, *P* < 0.001) and ASC (striatum: *F*_(1,11)_ = 10.593, *P* = 0.012; midbrain: *F*_(1,11)_ = 15.005, *P* = 0.005; Figures 8A2–A4,B2–B4). These results indicate that the Caspase-11 pathway is involved in TLR4-mediated METH-induced neuroinflammation *in vivo*.

## Discussion

Recent studies have demonstrated that METH can stimulate glial cells in the CNS, suggesting that neurotoxicity of METH could be partially due to neuroinflammation response, which includes astrocyte activation (Cisneros and Ghorpade, 2014; Borgmann and Ghorpade, 2015). In the present study, we report that TLR4 expression is increased after METH exposure *in vivo* and *in vitro*. Furthermore, we for the first time demonstrate that Caspase-11 plays an important role in METH-induced astrocyte stimulation and the NLRP3 inflammasome promotes the pro-inflammatory cytokine expression.

As the most abundant cell type in the brain, the primary roles of astrocytes include the protection of neurons, participation in myelination, expression of glutamate transporters, involvement of glutamate transport in the CNS, and a part of inflammation effects (Loftis and Janowsky, 2014; Zhang et al., 2017a). The results of inflammatory response are generally harmful, including regulation of microglia activation and the expression of inflammatory factors that could directly damage the neurons. Activation of astrocytes has a dual effect. Astrocyte hyperplasia can beneficially provide nutritional support for neurons and maintain the steady balance of the surrounding environment (Loftis and Janowsky, 2014; Singh and Abraham, 2017). While the hyperactive astrocytes may cause neuroinflammation, leading to an increase release of pro-inflammatory cytokines. In this study, we found that 2.0 mM of METH could increase the secretion of pro-inflammatory cytokines in astrocytes, and upregulate the expression of pro-inflammatory cytokines in the midbrain and striatum of C57BL/6 mice. Studies have shown that METH-treated astrocytes can produce neuroinflammation, oxidative stress and excitotoxicity, as well as morphological changes of the blood-brain barrier (Cisneros and Ghorpade, 2014; Loftis and Janowsky, 2014). However, the relationship between METH-induced astrocyte activation and production of proinflammatory cytokines remains to be investigated.

TLR4 is one type of immune pattern recognition receptors, which are the first defense of the body’s immune response (Krawczyk-Michalak et al., 2008; Matsunaga et al., 2011; Fang et al., 2013; Shirjang et al., 2017). Studies have reported that heroin can induce the activation of TLR4 on the cell surface, which in turn mediates the occurrence of inflammatory responses (Theberge et al., 2013). Here, we demonstrate that METH exposure induces the expression of TLR4 and TLR4 promotes the expression of NF-κB through both the TRIF signaling axis (Myd88 independent pathway) and the TIRAP/Myd88/IRAK4 signaling axis (Myd88 dependent pathway), leading to increased nuclear transcription of inflammatory cytokines. Specifically, following METH exposure, the expression levels of TRIF (the key protein of the Myd88 independent pathway) and Myd88 (the key protein of the Myd88 dependent pathway) were both increased; and these effects were normalized or significantly attenuated after inhibiting TLR4 expression by siRNA or TAK-242. Of note, the expression of NF-κB was also increased by METH, and this effect was significantly attenuated after inhibiting TLR4 expression by siRNA or TAK-242. These results suggest that the activation of NF-κB by METH may be through the TLR4 pathway.

The present study revealed the important role of TLR4 in astrocytes in METH-induced neuroinflammation, but TLR4 is not unique to astrocytes in the brain. In this study, we determined the co-localization of TLR4 expression in different brain cell types. We observed that METH treatment induced the expression of TLR4 and co-localization of TLR4 with GFAP (marker of astrocytes) and Iba1 (marker of microglia). We also observed that part of the NeuN (marker of neurons) expression was co-localized with TLR4 (methods and results provided in the Supplementary Figure S1). Studies of other neurological disease models have shown that TLR4 plays an important role in the pathogenesis of a variety of neuro-inflammation and is expressed in microglia and neurons (Wang et al., 2014; Baek et al., 2017; Chen et al., 2017; Lawrimore and Crews, 2017; Rocha Sobrinho et al., 2017). Our results suggest that METH treatment could also induce TLR4 activation in microglia and neurons. The role of microglia in METH-induced neuro-inflammation and the interactions of astrocytes with microglia and neurons in METH neurotoxicity are a direction of future research.

Caspase-11 mediates non-classical inflammatory responses and increases the release of pro-inflammatory cytokines, which has been demonstrated in other immune cell models (Viganò and Mortellaro, 2013; Coutermarsh-Ott et al., 2016). While previous studies mainly focused on macrophages (Viganò and Mortellaro, 2013), in this study, we used primary cultured astrocytes to mimic the response of astrocytes *in vivo* and to reveal the regulatory mechanism of Caspase-11. Previous studies of Caspase-11 mainly demonstrated that it was stimulated by LPS or other extracellular stimuli, resulting in the occurrence of inflammatory response (Kayagaki et al., 2011, 2013, 2015; Shi et al., 2015). Our studies showed that METH could also cause inflammatory response through Caspase-11. The expression of Caspase-11 decreased after silencing of TLR4, while the expression of TLR4 had no change after Caspase-11 silencing. Besides, the expression of Caspase-11 decreased after silencing of Myd88 and TRIF, indicating that the expression of Caspase-11 is activated by both Myd88-dependent and independent pathways of TLR4.

There is also an interesting finding in this study that the NLRP3 inflammasome is involved in Caspase-11-mediated neuroinflammation caused by METH in astrocytes. Caspase-11-dependent NLRP3 inflammasome activation was also reported during gram-negative bacterial infection (Rathinam et al., 2012). This indicates that METH might cause neuroinflammation through Caspase-11-mediated non-classical inflammatory pathway. However, we found that METH could only increase ASC and Caspase-1 expression, while NLRP3 expression was not changed. This may be due to that fact that a higher proportion of Caspase-1 is involved in the composition of the inflammasome complex (Wang et al., 1998; Man et al., 2017) and METH may primarily activate Caspase-1, rather than increase the overall expression of inflammasome NLRP3 itself, thereby inducing an increase in downstream inflammatory factors. Of note, there is also a classical pathway in which the increased expression of inflammatory factors can be induced by Caspase-1 directly (Youm et al., 2013; Man et al., 2017). Further studies are needed to determine whether Caspase-11 plays a role in the non-inflammasome NLRP3 pathway in the METH-induced astrocyte activation.

## Conclusion

**Figure 9:**
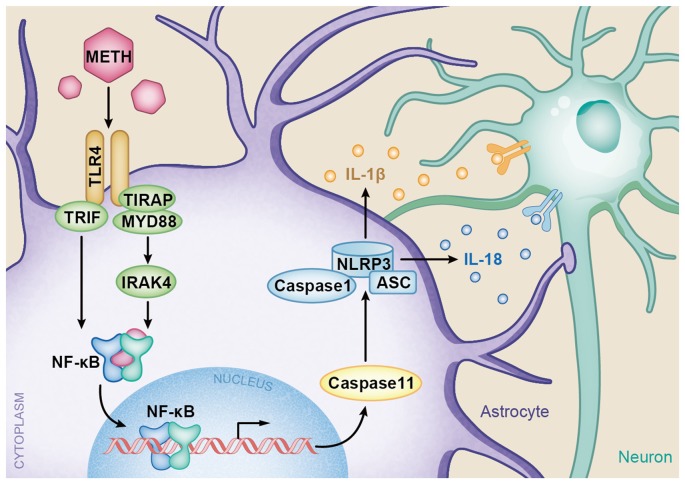
A schematic depicting the role of TLR4-NFkB-Caspase-11 signaling pathway in METH-induced astrocyte-related neuroinflammation. Briefly, TLR4 expression is increased following METH treatment. Increased TLR4 upregulates the expression of Myd88 and TRIF. The Myd88-dependent and Myd88-independent pathways upregulate the expression of transcription factor NF-κB. Then NF-κB elevates Caspase-11 expression, which mediates the inflammasome NLRP3 pathway by upregulating the expression of Caspase-1 and ASC. The increased NLRP3 inflammasomes induce the expression of pro-inflammatory cytokines IL-1β and IL-18.

In summary, the present study provides insights into the molecular mechanisms of TLR4 and Caspase-11-mediated METH-induced neuroinflammation in astrocytes. A schematic depicting the proposed mechanisms of METH-induced astrocyte neuroinflammation is provided in Figure 9. Specifically, METH can increase TLR4 and Caspase-11 expression *in vitro* and *in vivo*, and TLR4 plays an important role in astrocyte neuroinflammation induced by METH. The increased activation of the TLR4-regulated neuroinflammation is through both Myd88-dependent and independent pathways. As a transcription factor, NF-κB upregulates the expression of Caspase-11, resulting in upregulation of NLRP3 inflammasome, which mediates the increased expression levels of IL-1β and IL-18 after METH exposure. However, the underlying mechanisms of whether or not Caspase-11 could directly regulate pro-inflammatory cytokine expression need further research. Further studies are also needed to determine the exact mechanisms of neuronal apoptosis and autophagy regulated by astrocyte-related neuroinflammation in METH acute exposure models.

## Ethics Statement

All procedures involving animals were performed in accordance with the ethical standards of Ethics Committee of Nanfang Hospital, Southern Medical University and with the 1964 Helsinki Declaration and its later amendments or comparable ethical standards. This article does not contain any studies with humans performed by any of the authors.

## Availability of Data and Material

The authors declare that the data supporting the findings of this study are available within the article.

## Author Contributions

S-HD and D-FQ conducted all the experiments with the help of C-XC and SC. W-BX and HW designed the research. W-BX, S-HD, CL and ZL analyzed and interpreted the results. W-BX and S-HD wrote the manuscript with the help of ZL.

## Conflict of Interest Statement

The authors declare that the research was conducted in the absence of any commercial or financial relationships that could be construed as a potential conflict of interest.

## Abbreviations

ASC: apoptosis-associated speck-like protein containing CARD
CNS: central nervous system
DAB: 3,3′-diaminobenzidine
DAPI: 4′,6′-diamidino-2-phenylindole
DMEM: Dulbecco’s modified Eagle’s medium
FBS: Fetal bovine serum
IFN: Interferon
IL: Interleukin
IRAK: Interleukin-1 receptor-associated kinase
LPS: lipopolysaccharides
METH: Methamphetamine
NLRP3: nucleotide-binding oligomerization domain-like receptor family pyrin domain containing 3
PVDF: Polyvinylidenedifluoride
SDS-PAGE: Sodium dodecyl sulfate polyacrylamide gel
TLR4: Toll-like receptor 4
TRAM: TRIF-related adaptor molecule
Wed: Wedelolactone.
