# Supplementary material for: Toll-Like Receptor 4 Mediates Methamphetamine-Induced Neuroinflammation through Caspase-11 Signaling Pathway in Astrocytes
Source: Front Mol Neurosci. 2017 Dec 12;10:409. doi: 10.3389/fnmol.2017.00409 (PMC5733023; doi:10.3389/fnmol.2017.00409)
Supplement: Supplementary file 1 [file Data_Sheet_1.zip › Supplementary materials/Supplemental data.docx]

**Supplementary data**

**Toll-like receptor 4 mediates methamphetamine-induced neuroinflammation through Caspase-11 signaling pathway in astrocytes**

Si-Hao Du^a,1^, Dong-Fang Qiao^a,1^, Chuan-Xiang Chen^a^, Si Chen^a^, Chao Liu^b^, Zhoumeng Lin^c^, Huijun Wang^a*^, Wei-Bing Xie^a*^

a School of Forensic Medicine, Southern Medical University, Guangzhou 510515, China.b Guangzhou Forensic Science Institute, Guangzhou 510030, China; c Institute of Computational Comparative Medicine and Department of Anatomy and Physiology, College of Veterinary Medicine, Kansas State University, Manhattan, KS 66506, USA

1 These authors contributed equally to this work.

*Corresponding author at: School of Forensic Medicine, Southern Medical University, Guangzhou 510515, China. Telephone: +86-2062789044. E-mail addresses: hjwang@smu.edu.cn (Huijun Wang), xwb@smu.edu.cn (Wei-Bing Xie).

**Materials and methods**

**Materials**

Anti-TLR4 and Iba1 were purchased from Santa Cruz Biotechnology (California, USA). Anti-NeuN was purchased from Abcam (Cambridge, UK). Anti-GFAP was purchased from Arigobio laboratories. Anti-rabbit and mouse IgG (H+L), F(ab’)2 fragment (Alexa Fluor 555 conjugate) were purchased from the Cell Signaling Technology (Boston, MA, USA). Fluorescein (FITC)-conjugated goat anti-mouse and rabbit IgG were purchased from DingGuo (Beijing, China).

**Double immunofluorescence labeling**

To determine the co-localization of TLR4 expression in different cell types in mouse midbrain samples, we performed double immunofluorescence labeling on frozen sections of adult mouse midbrains. For immunolabeling, all incubation solutions were prepared using PBS supplemented with 10% normal goat serum and 0.05% Triton X-100. These antibodies were used together with DAPI nuclear labeling. The frozen tissue sections were incubated with blocking buffer (10% BSA in PBS) for 30 min at room temperature, with the primary antibody (anti-GFAP dilution of 1:500, anti-TLR4 dilution of 1:100, anti-Iba1 dilution of 1:10 and anti-NeuN dilution of 1:100) overnight at 4°C, and then with the secondary antibody for 1h at room temperature (FITC conjugated anti-mouse or rabbit IgG dilution of 1:50, Alexa Fluor 555 conjugated anti-mouse or rabbit IgG dilution of 1:200). Microphotographs were taken using fluorescence microscopy (A1+/A1R+; Nikon). All digital images were processed using the same settings to improve the contrast.

**Results**


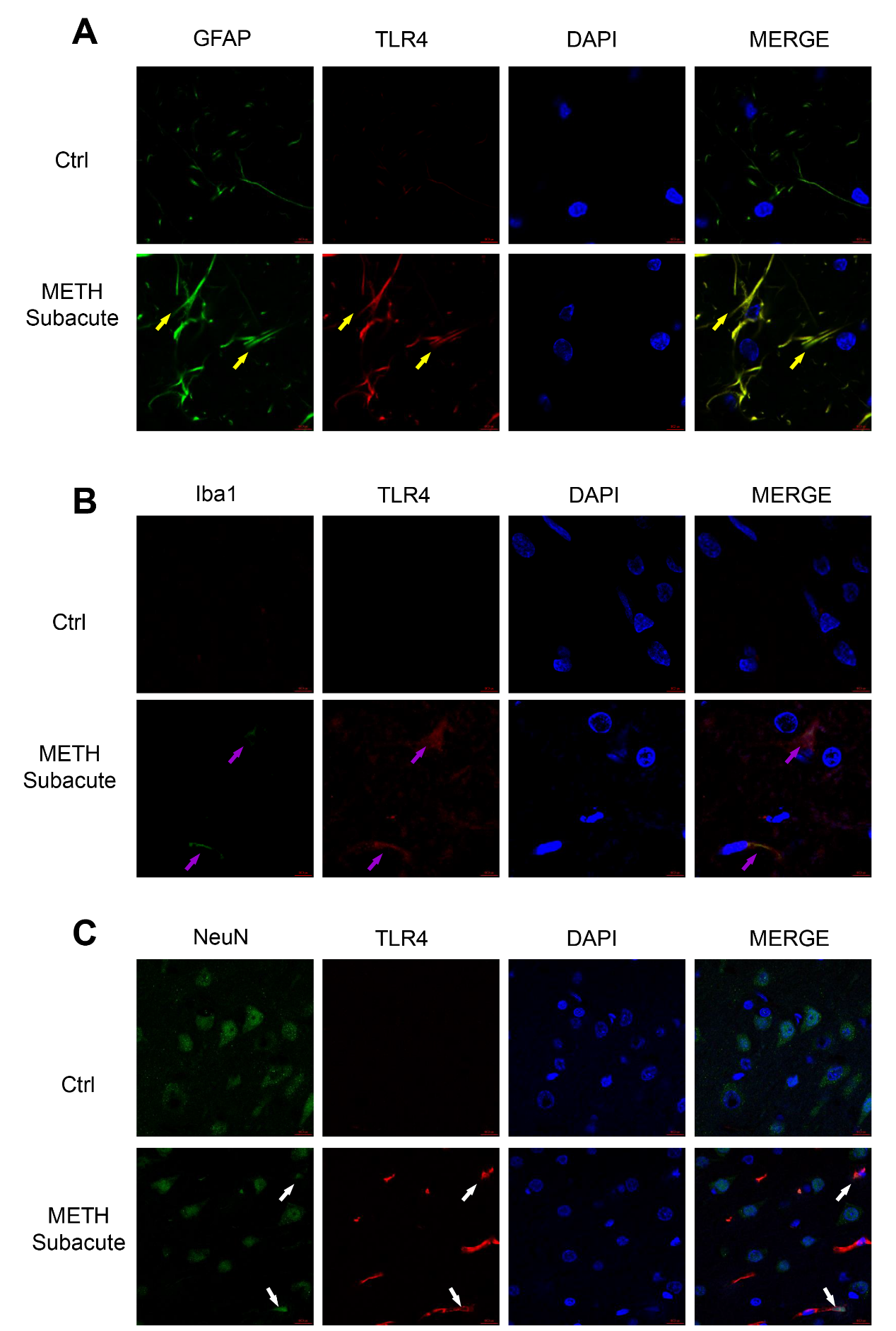


**Supplementary Fig.1** Male adult C57BL/6 mice were divided randomly into control and METH groups (n=5/group). Animals were injected intraperitoneally with saline or METH (15mg/kg/injection, 8 injections, at 12h intervals). Midbrain tissues were harvested at 24h after the last dosing. Immunolabelling and confocal imaging analysis showed elevated TLR4 expression in the midbrain of METH-exposed mice compared with controls. (A) Yellow arrow refers to the co-localization of TLR4 and GFAP (marker of astrocytes). (B) Purple arrow refers to the co-localization of TLR4 and Iba1 (marker of microglia). (C) White arrow refers to the co-localization of TLR4 and NeuN (marker of neurons).
