# Supplementary figures and images for: Toll-Like Receptor 4 Mediates Methamphetamine-Induced Neuroinflammation through Caspase-11 Signaling Pathway in Astrocytes
Source: Front Mol Neurosci. 2017 Dec 12;10:409. doi: 10.3389/fnmol.2017.00409 (PMC5733023; doi:10.3389/fnmol.2017.00409)

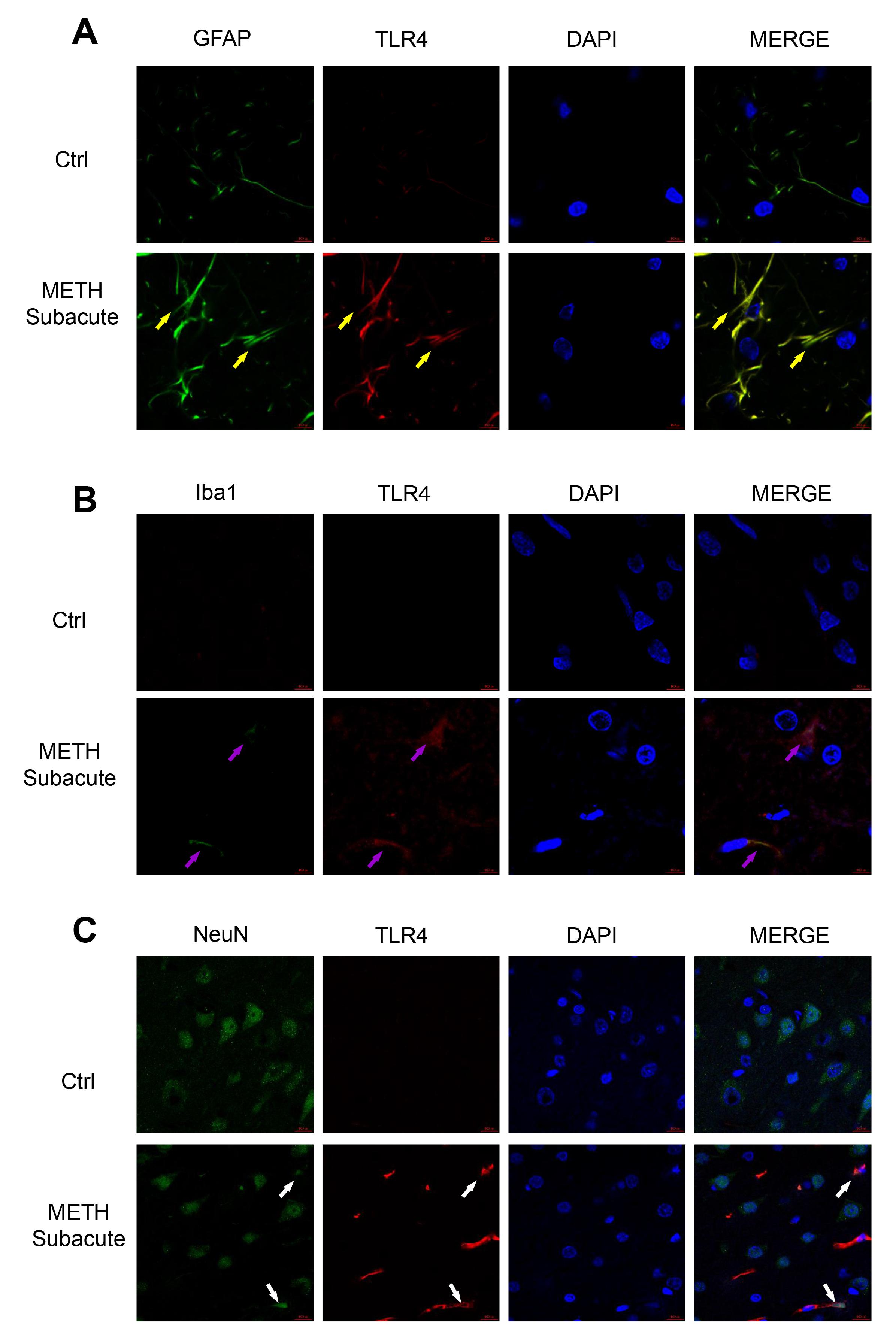

Supplement: Supplementary file 1 [file Data_Sheet_1.zip › Supplementary materials/Supplemental picture-1.tif]
